# Genome Sequences of 228 Shiga Toxin-Producing *Escherichia coli* Isolates and 12 Isolates Representing Other Diarrheagenic *E. coli* Pathotypes

**DOI:** 10.1128/genomeA.00718-14

**Published:** 2014-08-07

**Authors:** Eija Trees, Nancy Strockbine, Shankar Changayil, Satishkumar Ranganathan, Kun Zhao, Ryan Weil, Duncan MacCannell, Ashley Sabol, Amber Schmidtke, Haley Martin, Devon Stripling, Efrain M. Ribot, Peter Gerner-Smidt

**Affiliations:** a Division of Foodborne, Waterborne, and Environmental Diseases, Centers for Disease Control and Prevention, Atlanta, Georgia, USA; b Office of Infectious Diseases, Centers for Disease Control and Prevention, Atlanta, Georgia, USA

## Abstract

Shiga toxin-producing *Escherichia coli* (STEC) are a common cause for food-borne diarrheal illness outbreaks and sporadic cases. Here, we report the availability of the draft genome sequences of 228 STEC strains representing 32 serotypes with known pulsed-field gel electrophoresis (PFGE) types and epidemiological relationships, as well as 12 strains representing other diarrheagenic *E. coli* pathotypes.

## GENOME ANNOUNCEMENT

The rapidly decreasing cost of next-generation sequencing (NGS) will facilitate its application for real-time surveillance in the near future. PulseNet, the molecular subtyping network for food-borne disease surveillance, currently relies on pulsed-field gel electrophoresis (PFGE) to define clusters of illness (1). In order to use NGS as a primary method for cluster detection, a thorough understanding of the genetic diversity in the target population is needed. Shiga toxin-producing *Escherichia coli* (STEC) are among the pathogens tracked by PulseNet. In this report, we announce the availability of the draft sequences of a carefully selected set of STEC strains that should enable us to gain insights into the sequence diversity within an outbreak or a carrier state and among epidemiologically unrelated isolates within a serotype and between serotypes.

We sequenced 228 STEC strains representing 32 serotypes with known PFGE types and epidemiological relationships. The strain set included a total of 50 isolates from five outbreaks, 11 isolates from a long-term carrier, and epidemiologically unrelated strains. Twelve strains of other diarrheagenic *E. coli* pathotypes were included as outliers. Genomic DNA from each strain was isolated using the ArchivePure DNA cell/tissue kit (5Prime, Hamburg, Germany). All 240 strains were sequenced to a minimum depth of 100× with the HiSeq 2000 or GAIIx (Illumina, San Diego, CA, USA) using the TrueSeq DNA LT sample prep kit (Illumina) for DNA library preparation and 100-bp paired-end read chemistry. Additionally, 82 strains were sequenced with the PacBio RS system (Pacific Biosciences, Menlo Park, CA) using C2 chemistry and four single-molecule real-time (SMRT) cells per genome.

Raw read quality checks were performed on the 240 samples using FastQC (http://www.bioinformatics.bbsrc.ac.uk/projects/fastqc) and in-house Perl scripts/Java programs. Primary analysis for the Illumina data was performed using CLC Genomics Workbench 5.5.1 (Aarhus, Denmark). The raw read files for each sample were trimmed with length (minimum, 50 bp) and quality score (0.02) filters. The trimmed reads were assembled into contigs with specific parameter settings (length fraction, 0.8; similarity fraction, 0.8; minimum contig length, 450 bp), and assembly statistics were parsed out in a table format using in-house scripts. The PacBio data analysis was performed using the whole-genome sequencing (WGS) assembler toolkit (2). Error correction of the filtered subreads was performed with the paired-end Illumina data (~60× data was used) using the WGS toolkit PacBioToCA script, followed by *de novo* assembly using the runCA script. The best assembly for each of these 82 samples was chosen based on the number of contigs, *N*_50_ value, and genome length.

The average genome size for the sequenced strains was 5,282,291 bp (range, 4,527,885 to 5,712,627). For the 240 Illumina assemblies, the average number of contigs was 211 (range, 68 to 465), and the average *N*_50_ was 128,850 (range, 26,435 to 230,877). For the 82 PacBio hybrid assemblies, the average number of contigs was 207 (range, 31 to 207), and the average *N*_50_ was 172,854 (range, 31,094 to 1,414,730).

### Nucleotide sequence accession numbers.

The draft genome sequences for these 240 diarrheagenic *E. coli* strains have been deposited in DDBJ/ENA/GenBank under the accession numbers listed in Table 1.

**Table 1:** NCBI accession numbers for 240 *E. coli* draft genomes

Strain ID	Serotype	NCBI accession no.
00-3279	O78:H12	JFBE00000000
01-3076	O111:NM	JFGU00000000
01-3147	O45:H2	JHOA00000000
02-3012	O81:NM	JHNZ00000000
02-3404	O28ac:NM	JHNY00000000
03-3227	O121:H19	JHNX00000000
03-3269	O174:H21	JHNW00000000
03-3458	O119:H4	JHNV00000000
03-3484	O111:NM	JHNU00000000
03-3500	O26:H11	JHNT00000000
04-3023	O103:H11	JHOD00000000
04-3038	O174:H8	JHOC00000000
04-3211	O111:NM	JHNS00000000
05-3646	026:H11	JHOE00000000
06-3003	O121:H19	JHNR00000000
06-3256	O118:H16	JHNQ00000000
06-3325	O69:H11	JHNP00000000
06-3464	O26:H11	JHNO00000000
06-3484	O145:NM	JHNN00000000
06-3501	O79:H7	JHNM00000000
06-3555	O55:H7	JHNL00000000
06-3612	O118:H16	JHNK00000000
06-3691	O91:H14	JHNJ00000000
06-3745	O157:H7	JHNI00000000
06-3822	O121:H19	JHNH00000000
06-4039	O157:H7	JHNG00000000
07-3091	O157:H7	JHNF00000000
07-3391	O157:H7	JHNE00000000
07-4224	O113:H21	JHOB00000000
07-4281	O69:H11	JHLA00000000
08-3037	O157:H7	JHKZ00000000
08-3527	O157:H7	JHKY00000000
08-3651	O118:H16	JHKX00000000
08-4169	O157:H7	JHKW00000000
08-4270	O145:NM	JHKV00000000
08-4487	O111:NM	JHKU00000000
08-4529	O157:H7	JHHI00000000
08-4540	O157:NM	JHHH00000000
08-4661	O69:H11	JHHG00000000
2009C-3227	O91:H14	JHHF00000000
2009C-3279	O103:H2	JHHE00000000
2009C-3292	O145:H28	JHHD00000000
2009C-3299	O121:H7	JHHC00000000
2009C-3307	O123:H11	JHHB00000000
2009C-3601	O69:H11	JHHA00000000
2009C-3612	O26:H11	JHGZ00000000
2009C-3686	O45:H2	JHGY00000000
2009C-3689	O26:H11	JHGX00000000
2009C-3745	O91:NM	JHGW00000000
2009C-3996	O26:H11	JHGV00000000
2009C-4006	O111:NM	JHGU00000000
2009C-4050	O121:H19	JHGT00000000
2009C-4052	O111:NM	JHGS00000000
2009C-4126	O111:H8	JHGR00000000
2009C-4258	O157:H7	JHGQ00000000
2009C-4446	O118:H16	JHGP00000000
2009C-4646	O91:H21	JHGO00000000
2009C-4659	O121:H19	JHGN00000000
2009C-4747	O26:H11	JHGM00000000
2009C-4750	O121:H19	JHGL00000000
2009C-4760	O26:H11	JHGK00000000
2009C-4780	O45:H2	JHGJ00000000
2009C-4826	O26:H11	JHGI00000000
2009EL1302	O121:H19	JHGH00000000
2009EL1412	O121:H19	JHGG00000000
2009EL1449	O157:H7	JHGF00000000
2009EL1705	O157:H7	JHGE00000000
2009EL1913	O157:H7	JHGD00000000
2009EL2109	O157:H7	JHGC00000000
2009EL-2169	O111:H8	JHGB00000000
2010C-3051	O26:H11	JHGA00000000
2010C-3053	O111: NM	JHFZ00000000
2010C-3214	O103:H11	JHFY00000000
2010C-3472	O26:H11	JHFX00000000
2010C-3507	O145:NM	JHFW00000000
2010C-3508	O145:NM	JHFV00000000
2010C-3509	O145:NM	JHFU00000000
2010C-3510	O145:NM	JHFT00000000
2010C-3511	O145:NM	JHFS00000000
2010C-3516	O145:NM	JHFR00000000
2010C-3517	O145:NM	JHFQ00000000
2010C-3518	O145:NM	JHFP00000000
2010C-3521	O145:NM	JHFO00000000
2010C-3526	O145:NM	JHFN00000000
2010C-3609	O121:H19	JHFM00000000
2010C-3794	O121:H19	JHFL00000000
2010C-3840	O121:H19	JHFK00000000
2010C-3871	O26:H11	JHFJ00000000
2010C-3876	O45:H2	JHFI00000000
2010C-3902	O26:H11	JHFH00000000
2010C-3977	O111:NM	JHFG00000000
2010C-4086	O111:NM	JHFF00000000
2010C-4221	O111:NM	JHFE00000000
2010C-4244	O26:H11	JHFD00000000
2010C-4254	O121:H19	JHFC00000000
2010C-4347	O26:NM	JHFB00000000
2010C-4430	O26:H11	JHND00000000
2010C-4433	O103:H2	JHNC00000000
2010C-4529	O103:H25	JHNB00000000
2010C-4557C2	O145:NM	JHNA00000000
2010C-4558	O177:NM	JHMZ00000000
2010C-4592	O111:NM	JHMY00000000
2010C-4622	O111:NM	JHMX00000000
2010C-4715	O111:NM	JHMW00000000
2010C-4732	O121:H19	JHMV00000000
2010C-4735	O111:NM	JHMU00000000
2010C-4746	O111:NM	JHMT00000000
2010C-4788	O26:NM	JHMS00000000
2010C-4799	O111:NM	JHMR00000000
2010C-4818	O111:NM	JHMQ00000000
2010C-4819	O26:H11	JHMP00000000
2010C-4824	O121:H19	JHMO00000000
2010C-4834	O26:H11	JHMN00000000
2010C-4874	O165:H25	JHMM00000000
2010C-4966	O121:H19	JHML00000000
2010C-4979C1	O157:H7	JHMK00000000
2010C-4989	O121:H19	JHMJ00000000
2010C-5028	O26:H11	JHMI00000000
2010C-5034	O153:H2	JHMH00000000
2010EL1058	O121:H19	JHMG00000000
2010EL-1699	O26:H11	JHMF00000000
2010EL-2044	O157:H7	JHME00000000
2010EL-2045	O157:H7	JHMD00000000
2011C-3072	O121:H19	JHMC00000000
2011C-3108	O121:H19	JHMB00000000
2011C-3170	O111:NM	JHMA00000000
2011C-3216	O121:H19	JHLZ00000000
2011C-3270	O26:H11	JHLY00000000
2011C-3282	O26:H11	JHLX00000000
2011C-3362	O111:NM	JHLW00000000
2011C-3387	O26:H11	JHLV00000000
2011C-3453	O111:H8	JHLU00000000
2011C-3500	O121:H19	JHLT00000000
2011C-3506	O26:H11	JHLS00000000
2011C-3537	O121:H19	JHLR00000000
2011C-3573	O111:NM	JHLQ00000000
2011C-3602	O156:H25	JHLP00000000
2011C-3632	O111:NM	JHLO00000000
2011C-3655	O26:H11	JHLN00000000
2011C-3679	O111:NM	JHLM00000000
2011C-3750	O103:H2	JHLL00000000
2011EL-1107	O157:H7	JHLK00000000
2011EL-1675A	O104:H4	JHLJ00000000
2011EL-2090	O157:H7	JHLI00000000
2011EL-2091	O157:H7	JHLH00000000
2011EL-2092	O157:H7	JHLG00000000
2011EL-2093	O157:H7	JHLF00000000
2011EL-2094	O157:H7	JHLE00000000
2011EL-2096	O157:H7	JHLD00000000
2011EL-2097	O157:H7	JHLC00000000
2011EL-2098	O157:H7	JHLB00000000
2011EL-2099	O157:H7	JHKT00000000
2011EL-2101	O157:H7	JHKS00000000
2011EL-2103	O157:H7	JHKR00000000
2011EL-2104	O157:H7	JHKQ00000000
2011EL-2105	O157:H7	JHKP00000000
2011EL-2106	O157:H7	JHKO00000000
2011EL-2107	O157:H7	JHKN00000000
2011EL-2108	O157:H7	JHKM00000000
2011EL-2109	O157:H7	JHKL00000000
2011EL-2111	O157:H7	JHKK00000000
2011EL-2112	O157:H7	JHKJ00000000
2011EL-2113	O157:H7	JHKI00000000
2011EL-2114	O157:H7	JHKH00000000
2011EL-2286	O157:H7	JHKG00000000
2011EL-2287	O157:H7	JHKF00000000
2011EL-2288	O157:H7	JHKE00000000
2011EL-2289	O157:H7	JHKD00000000
2011EL-2290	O157:H7	JHKC00000000
2011EL-2312	O157:H7	JHKB00000000
2011EL-2313	O157:H7	JHKA00000000
94-3025	0104:H21	JHJZ00000000
98-3133	O157:H16	JHJY00000000
99-3124	O86:H34	JHJX00000000
99-3165	O6:H16	JHJW00000000
E2539C1	O25:NM	JHJV00000000
F5656C1	O6:H16	JHJU00000000
F6142	O157:H7	JHJT00000000
F6627	0111:H8	JHJS00000000
F6714	0121:H19	JHJR00000000
F6749	O157:H7	JHJQ00000000
F6750	O157:H7	JHJP00000000
F6751	O157:H7	JHJO00000000
F7350	O157:H7	JHJN00000000
F7377	O157:H7	JHJM00000000
F7384	O157:H7	JHJL00000000
F7410	O157:H7	JHJK00000000
F9792	O169:H41	JHJJ00000000
G5303	O157:H7	JHJI00000000
H2495	O157:H7	JHJH00000000
H2498	O157:H7	JHJG00000000
K1420	O157:H7	JHJF00000000
K1516	O15:H18	JHJE00000000
K1792	O157:H7	JHJD00000000
K1793	O157:H7	JHJC00000000
K1795	O157:H7	JHJB00000000
K1796	O157:H7	JHJA00000000
K1845	O157:H7	JHIZ00000000
K1921	O157:H7	JHIY00000000
K1927	O157:H7	JHIX00000000
K2188	O157:H7	JHIW00000000
K2191	O157:H7	JHIV00000000
K2192	O157:H7	JHIU00000000
K2324	O157:H7	JHIT00000000
K2581	O157:H7	JHIS00000000
K2622	O157:H7	JHIR00000000
K2845	O157:H7	JHIQ00000000
K2854	O157:H7	JHIP00000000
K4396	O157:H7	JHIO00000000
K4405	O157:H7	JHIN00000000
K4406	O157:H7	JHIM00000000
K4527	O157:H7	JHIL00000000
K5198	O121:H19	JHIK00000000
K5269	O121:H19	JHIJ00000000
K5418	O157:H7	JHII00000000
K5448	O157:H7	JHIH00000000
K5449	O157:H7	JHIG00000000
K5453	O157:H7	JHIF00000000
K5460	O157:H7	JHIE00000000
K5467	O157:H7	JHID00000000
K5602	O157:H7	JHIC00000000
K5607	O157:H7	JHIB00000000
K5609	O157:H7	JHIA00000000
K5806	O157:H7	JHHZ00000000
K5852	O157:H7	JHHY00000000
K6590	O157:H7	JHHX00000000
K6676	O157:H7	JHHW00000000
K6687	O157:H7	JHHV00000000
K6722	O111:NM	JHHU00000000
K6723	O111:NM	JHHT00000000
K6728	O111:NM	JHHS00000000
K6890	O111:NM	JHHR00000000
K6895	O111:NM	JHHQ00000000
K6897	O111:NM	JHHP00000000
K6898	O111:NM	JHHO00000000
K6904	O111:NM	JHHN00000000
K6908	O111:NM	JHHM00000000
K6915	O111:NM	JHHL00000000
K7140	O157:H7	JHHK00000000
F8704-2	O39:NM	JHHJ00000000
